# Satisfaction in Romantic Relationships: The Role of Body Appreciation, Sexual Esteem and Sexual Assertiveness

**DOI:** 10.3390/bs15121730

**Published:** 2025-12-15

**Authors:** Marco Rizzo, Camilla Matera, Daniela Caso, Anna Rosa Donizzetti, Caterina Grano, Amanda Nerini, Chiara Rollero

**Affiliations:** 1 Department of Theoretical and Applied Sciences, eCampus University, 22060 Novedrate, Italy; 2 Department of Psychology, University of Florence, 50135 Florence, Italy; camilla.matera@unifi.it (C.M.); amanda.nerini@unifi.it (A.N.); 3 Department of Humanities, University of Naples Federico II, 80138 Naples, Italy; caso@unina.it (D.C.); donizzet@unina.it (A.R.D.); 4 Department of Psychology, University of Rome La Sapienza, 00185 Rome, Italy; caterina.grano@uniroma1.it; 5 Department of Psychology, University of Turin, 10124 Turin, Italy; chiara.rollero@unito.it

**Keywords:** sexual assertiveness, body appreciation, sexual esteem, relationship satisfaction, individuals in heterosexual relationships

## Abstract

Romantic relationship satisfaction is widely recognized as a foundational contributor to both psychological and physical well-being. However, research on its association with other key constructs, such as body appreciation, sexual esteem and sexual assertiveness, is still lacking, especially in reference to romantic relationships. The present study investigates the interplay between body appreciation, sexual esteem, and sexual assertiveness, and their combined influence on relationship satisfaction in individuals in heterosexual relationships in Italy. Participants were 473 adults (50.1% men, 49.7% women, 0.2% other; age range 18–49). Structural equation modeling revealed that while body appreciation was indirectly associated with sexual assertiveness, it significantly predicted sexual esteem. Sexual assertiveness, but not sexual esteem, was positively associated with relationship satisfaction. No significant gender differences were found in the structural relationships among the variables. These findings underscore a process through which sexual assertiveness and body appreciation can enhance relational well-being and inform educational programs aimed at promoting healthy romantic relationships.

## 1. Introduction

Romantic relationship satisfaction is conceptualized as a subjective evaluation of relationship quality and of positivity toward one’s partner (Fallis et al., 2016; Keizer, 2014). Empirical studies highlight the importance of relationship satisfaction as a strong predictor of individual well-being and longevity (Bühler et al., 2020; Cassepp-Borges et al., 2025; Freihart et al., 2023; Fuller-Iglesias, 2015). Indeed, a successful and fulfilling romantic relationship is widely recognized as a foundational contributor to both psychological and physical well-being. Those who report high satisfaction in their romantic partnerships tend to be happier, enjoy better health, and live longer (Ruffieux et al., 2014). Such individuals also typically exhibit lower levels of stress, anxiety, and depressive symptoms, as well as higher life satisfaction and overall well-being (Roberson et al., 2018; Du Rocher Schudlich et al., 2011). Recent longitudinal analyses and meta-analyses reinforce these findings, demonstrating that relationship quality significantly predicts mental health trajectories over time, with stronger partnerships being associated with more favorable psychological outcomes (Bühler et al., 2020; Downward et al., 2022). Additionally, Cassepp-Borges et al. (2025) found that the influence of some love components—particularly passion and intimacy—on relationship satisfaction intensifies across relationship stages, further underscoring the dynamic interplay between emotional bonds and well-being.

Satisfaction in romantic relationships is influenced by a variety of factors. Most studies have focused on individual psychological dimensions, such as emotion regulation, commitment, internal working models of attachment styles, and personality traits (e.g., Anglim et al., 2020; Bühler et al., 2020; Cahill et al., 2020; Choi & Lim, 2023; Neto et al., 2023; Fu et al., 2025). Other studies have examined how certain events can impact relationship satisfaction and disrupt couples, such as when one partner becomes ill and the other provides care (e.g., Carberry et al., 2025; Fee et al., 2020; Rollero, 2016; Warrier et al., 2022).

Studies focusing on psychosocial dimensions are less common and emphasize the pivotal role of sexuality in fostering relationship satisfaction (Birnbaum & Muise, 2025; Dobson et al., 2020; Mallory, 2022). In some cases, it is even more important than other key factors, such as intimacy, agreement, and independence (Fallis et al., 2016; Hassebrauck & Fehr, 2002). In a romantic relationship, sexuality can refer not only to sexual frequency but also to the ability to communicate desires and needs to a partner in order to be satisfied. The ability to communicate with one’s partner about sexuality has been shown to be positively associated with both sexual and relational satisfaction (Frederick et al., 2017; Mallory, 2022). Frequent sexual communication has also been linked to increased orgasm frequency in women (Jones et al., 2018). Furthermore, communicating with one’s partner can serve as an effective strategy for addressing desire discrepancy in a relationship, thus preserving relationship quality (Gunst et al., 2024; Nickull et al., 2025; Vowels & Mark, 2020).

Sexual communication can be understood as one aspect of a broader concept, namely sexual assertiveness. Sexual assertiveness is defined as the ability to voluntarily initiate or refuse unwanted sexual activity, as well as the capacity to discuss one’s sexual history with a sexual partner and negotiate contraceptive use (Loshek & Terrell, 2015; Moyano et al., 2021). While the literature highlights the psychological and psychosocial benefits of sexual assertiveness (Boket et al., 2016; Brassard et al., 2015; Loshek & Terrell, 2015; Moyano et al., 2021; Zhang et al., 2022), to the best of our knowledge, its impact on couple satisfaction has not yet been specifically investigated in stable relationships.

### 1.1. Sexual Assertiveness and Relationship Satisfaction

Numerous studies have demonstrated a positive association between sexual assertiveness and various indicators of psychosocial well-being, such as interpersonal satisfaction, self-expression, self-efficacy, self-esteem, and overall mental health (Boket et al., 2016; Gil-Llario et al., 2022; López Alvarado et al., 2020; Moyano et al., 2021). These associations have been corroborated by recent empirical studies. For example, a study with university students in Germany demonstrated that higher levels of sexual assertiveness predict enhanced sexual satisfaction, sexual functioning, and relational well-being (Gimmel et al., 2024). Concurrently, longitudinal network analyses have shown a bidirectional relationship between sexual satisfaction and relational intimacy, thereby suggesting that sexual assertiveness may serve a mediating role in sustaining healthy romantic partnerships (Beaulieu et al., 2023). Conversely, deficits in sexual assertiveness have been linked to a spectrum of adverse outcomes, such as engagement in high-risk sexual behaviors, increased vulnerability to sexually transmitted infections, unintended pregnancies, experiences of humiliation and blame, conflictual intimate relationships, diminished sexual satisfaction, and heightened risk of sexual victimization (Couture et al., 2023; Krahé & Berger, 2017; Oesterle et al., 2022; Rollero et al., 2023; Sánchez-Fuentes et al., 2025; Schuster et al., 2023). While sexual assertiveness has been widely demonstrated to be an important aspect of a person’s sexual life, few studies have investigated what factors might promote the growth of sexual assertiveness.

### 1.2. The Role of Body Appreciation and Sexual Esteem on Sexual Assertiveness

With regard to variables that may influence sexual assertiveness, a recent systematic review (López Alvarado et al., 2020) has focused on several aspects, such as demographic variables (e.g., age and education), sexual experience (e.g., type of partnership), and cultural context issues (e.g., gender stereotypes). Psychological factors also play a key role, particularly one’s relationship with and appreciation of one’s body. Body appreciation is defined as accepting, holding favorable opinions toward, and respecting the body (Tylka & Wood-Barcalow, 2015). As shown in a recent meta-analysis, body appreciation is associated with adaptive psychological constructs related to sexuality (Linardon et al., 2022). However, although body appreciation constitutes a central element in promoting satisfying sexuality, only a limited number of studies have specifically investigated the relationship between body appreciation and sexual assertiveness. One of them examined the association between body appreciation and sexual agency among a US community sample of 355 heterosexual women with different marital statuses aged 18–40 years (Grower & Ward, 2018). The results demonstrated that higher levels of body appreciation were associated with greater sexual assertiveness, as well as with other aspects related to sexual well-being (e.g., sexual satisfaction, sexual pleasure, and increased confidence in negotiating condom use; Grower & Ward, 2018). Subsequently, Dai et al. (2021) investigated the role of body appreciation on sexual assertiveness and showed that it was positively associated with sexual assertiveness. The sample population comprised exclusively Chinese female college students, irrespective of their marital status.

Thus, these studies confirm, at least in the college population, the positive influence of body appreciation on assertiveness. On the other hand, the literature on body appreciation (e.g., Matera et al., 2024; Robbins & Reissing, 2018; Tylka & Wood-Barcalow, 2015; Wu & Zheng, 2021) showed that body appreciation is positively linked to self-evaluation and self-concept, including self-esteem in the sexual domain.

Sexual esteem refers to an individual’s positive evaluation of themselves as a sexual being, encompassing perceptions of sexual worth, competence, and confidence in expressing and engaging in sexuality (Brassard et al., 2015; Heinrichs et al., 2009). It is considered a key dimension of sexual self-concept and sexual well-being (Deutsch et al., 2014). Literature is controversial concerning the role that sexual esteem plays in sexual assertiveness. On the one hand, sexual esteem and sexual assertiveness are considered as two aspects closely and positively interrelated in fostering further general aspects of sexuality, such as sexual functioning, especially in women (Chesli et al., 2024) or sexual compliance (Nickull et al., 2025). On the other hand, the literature makes clear that sexual assertiveness may be a potential mediator in the relationship between sexual esteem and sexual satisfaction, as higher sexual self-esteem increases levels of sexual assertiveness (May & Johnston, 2022; Ménard & Offman, 2009).

By uniting the literature on body appreciation, sexual esteem and assertiveness, we posit that body appreciation can significantly influence sexual assertiveness, both directly and via the mediation of sexual esteem. Indeed, individuals who accept and value their bodies tend to exhibit reduced self-criticism and a more open expression of their sexual desires and boundaries. In other words, sexual self-esteem, derived from body appreciation, can serve as a tangible component that assists an individual in perceiving themselves in a favorable manner within the sexual domain. This perception can influence the manner in which one’s sexuality is expressed, aligning with the tenets of sexual respect and autonomy intrinsic to sexual assertiveness.

### 1.3. Current Study

Although previous studies have clarified the associations among body appreciation, sexual esteem, and sexual assertiveness (e.g., Grower & Ward, 2018; Linardon et al., 2022; May & Johnston, 2022), to the best of our knowledge, no study has simultaneously examined the interrelations among all three constructs. Moreover, the impact of these factors on individuals’ satisfaction with their relationships has not been specifically investigated yet.

The primary aim of the present study was to explore the factors associated with relationship satisfaction in a sample of individuals currently involved in a heterosexual romantic relationship. Specifically, we examined the role of body appreciation, sexual assertiveness, and sexual esteem. We hypothesized that body appreciation would be positively and directly associated with sexual assertiveness (Dai et al., 2021; Grower & Ward, 2018) and indirectly associated through the mediation of sexual esteem (May & Johnston, 2022; Ménard & Offman, 2009). In turn, relationship satisfaction was expected to be positively associated with sexual esteem (May & Johnston, 2022) and sexual assertiveness (Beaulieu et al., 2023; Gimmel et al., 2024). In addition, the present study was guided by extant literature recommendations (e.g., Moradi & Huang, 2008; Rollero, 2022) with the objective of empirically investigating whether these associations differ significantly between men and women in heterosexual relationships. This investigation was undertaken in order to avoid the assumption of equivalence among these constructs between men and women.

Therefore, the following hypotheses were proposed:

**H1.**  *Body appreciation will be positively associated with sexual assertiveness.*

**H2.**  *Body appreciation will be positively associated with sexual esteem.*

**H3.**  *Sexual esteem will mediate the relationship between body appreciation and sexual assertiveness.*

**H4.**  *Sexual esteem will be positively associated with relationship satisfaction.*

**H5.**  *Sexual assertiveness will be positively associated with relationship satisfaction.*

**H6.**  *No gender differences will emerge in the structural paths after establishing measurement invariance.*

## 2. Materials and Methods

### 2.1. Participants and Procedures

This work is part of a larger study whose main aim was to investigate the role of compassionate skills in improving body appreciation, which in turn would improve health-related outcomes. This research was funded by the Italian Ministry of University and Research—MUR NextGenerationEU (Fostering compassion abilities: potential benefits on body image and health-related outcomes; B53D23019650006). The study was approved by the Ethical Committee of the University to which some of the authors were affiliated (University of Turin, Italy—protocol number 0639477, 12 July 2023). Participants were recruited using snowball sampling from students in their first year of psychology. Various channels were used, such as social media and word-of-mouth, and participants were provided with a link through which they agreed to participate in the study. Each online self-report questionnaire was anonymous, and informed consent was given before the start of the study. Participants were informed that their participation was voluntary and that they could withdraw from the study at any time. No compensation was offered for participation. The questionnaire took approximately 20 min to complete.

For this paper, individuals who declared that they were in a romantic relationship at the time of the study were chosen as participants. Since non-heterosexual individuals constituted a clear minority, we selected only people who described themselves as heterosexual (N = 518). From this sample, we excluded those who did not complete the entire questionnaire and 12 participants who were identified as outliers through the multivariate test using the Mahalanobis distance (*D*^2^). The Mahalanobis distances were compared against the critical value of the χ^2^ distribution at the significance level *p* < 0.001 (df = 40).

The final study sample comprised 473 participants, balanced between men (50.1%) and women (49.7%), while 0.2% of participants answered “other”. The mean age was 22.83 years (SD = 5.49, age range 18–49). Most participants were Italian citizens (96.8%) and resided in central Italy (81.8%). The educational level was generally high: 72.3% had completed high school, and 13.1% had a university degree. The high proportion of individuals with a high school diploma was partially explained by the large number of students (57.5%), followed by participants who were employed either full-time, part-time, or occasionally (37.4%).

Regarding the relationship duration, the sample showed high variability, ranging from less than 1 year to 30 years. The average relationship length was 3.00 years (SD = 4.00).

### 2.2. Measures

For the present study, the following measures were used. When available, Italian validated versions of the instruments were used; otherwise, an Italian adaptation was provided through a back-translation procedure.

The Sexual Assertiveness Questionnaire (SAQ; Loshek & Terrell, 2015): This included a reduced version with 13 of the 18 items measuring sexual assertiveness on the following two dimensions considered pertinent for the larger study: the ability to initiate and communicate about sex (SAQ-COM, 8 items, e.g., ‘I let my partner know if I want to have sex’) and the ability to refuse unwanted sex (SAQ-REF, 5 items, e.g., ‘I refuse to have sex if I do not want to’). The items were rated on a 7-point Likert-type scale ranging from ‘completely false’ (1) to ‘completely true’ (7). Higher scores on each subscale imply high levels of sexual assertiveness.

The Body Appreciation Scale (BAS-2; Tylka & Wood-Barcalow, 2015; Italian validation version: Casale et al., 2021): This included 10 items that measure a unidimensional perception of positive body image (e.g., ‘I feel good about my body’). The items were rated on a 5-point Likert-type scale ranging from ‘never’ (1) to ‘always’ (5). Higher scores imply high levels of body appreciation.

The Sexual Esteem Subscale of the Multidimensional Sexuality Scale (SEX-EST; Snell et al., 1993): This included 5 items that measure a unidimensional sexual esteem (e.g., ‘I am confident about myself as a sexual partner’). The items were rated on a 5-point Likert-type scale ranging from ‘does not represent me at all’ (1) to ‘represents me very much’ (5). Higher scores imply high levels of sexual esteem.

The Dyadic-Familiar Relationship Satisfaction Scale (DFRS) (Raffagnino & Matera, 2015; Italian validation version): This included 9 items assessing the degree of satisfaction in the current dyadic relationship across different areas (e.g., ‘Way in which my wants and needs have been met in the current relationship’). The items were rated on a 5-point Likert-type scale ranging from ‘not satisfied at all’ (0) to ‘completely satisfied’ (4). Higher scores imply high levels of relationship satisfaction.

Finally, a list of sociodemographic items was administered, including sex, age, educational level, and romantic relationship duration.

### 2.3. Data Analysis

The analyses were performed with SPSS version 29.0 and MPLUS 8 (Muthén & Muthén, 2017). *t*-tests were performed to assess mean differences, controlling for the assumption of homoscedasticity of the variance, and the adjusted *p*-value was considered according to the number of hypotheses (Bonferroni correction). Cohen’s d was used to determine the effect size of the significant *t*-tests. Bivariate correlations were performed to analyze the relationships between the study variables. Confirmatory factor analysis (CFA) was performed to assess the measurement model of the scales. To test the hypotheses, a structural equation model (SEM) with mediation was estimated. Relationship duration and the gender (recoded in the dummy variable men and women) were used as control variables. Then, to examine for differences between women and men, measurement and structural invariance were tested. CFAs were tested for all instruments used in this study, both those already validated in an Italian version (BAS and DFRS) and those not yet validated in an Italian context. Testing the psychometric properties of the scale is a fundamental step in testing hypotheses in an SEM with latent variables (Kline, 2015). In addition, Cronbach’s alpha (α) was calculated to confirm reliability for each measure in line with original and validation studies.

All the analyses (CFA, SEM) were performed using the Asparouhov and Muthén (2010) mean- and variance-adjusted ML method of estimation (MLMV). This estimation method provides satisfactory accuracy in standard errors and controls Type I error in the presence of non-normal data, and it represents a reliable estimator, especially with large sample, as in the present study (Li, 2016). Furthermore, when ordinal variables have five or more response categories, as in the present study, the use of this robust estimator yields parameter estimates similar to those obtained with continuous indicators (Rhemtulla et al., 2012).

The model fit for CFA and SEM was assessed using three criteria: root mean square error of approximation (RMSEA) ≤ 0.080; comparative fit index (CFI) ≥ 0.900; and standardized root mean square residual (SRMR) ≤ 0.080 (Browne & Cudeck, 1992; Hu & Bentler, 1999). Modification indices were used in the case of problematic model fit, provided that their content was theoretically coherent.

To assess measurement invariance between men and women, three increasingly restrictive models were estimated: configural (all parameters were freely estimated across groups), metric (loadings were constrained to be equal across groups), and scalar (loadings and intercepts were constrained to be equal across groups; Putnick & Bornstein, 2016; Vandenberg & Lance, 2000). When configural, metric, or scalar invariance failed to meet the required criteria for acceptable model fit, partial invariance was established. This was achieved by systematically identifying specific indicator items that were found to be non-invariant (i.e., those whose equality constraint significantly degraded model fit, as indicated by modification indices or stepwise fit comparisons; Putnick & Bornstein, 2016). Full or partial measurement invariance is necessary for each measure to assess the equivalence between men and women, which then allows for testing of structural invariance. Structural invariance was assessed by comparing an unconstrained model (M1; baseline) with a constrained model (M2; structural) in which all relationships were constrained to be equal for women and men.

The main criterion to assess a lack of invariance was a ΔCFI ≤ −0.005 and at least one of the two following criteria: ΔRMSEA ≥ 0.010 and ΔSRMR ≥ 0.025 for metric invariance and ≥0.005 for scalar invariance (Chen, 2007). Furthermore, an additional criterion was considered for Δχ^2^: the lack of invariance was assumed when the ratio between Δχ^2^ and Δdegrees of freedom (Δdf) was >3 (Schermelleh-Engel et al., 2003).

A bootstrap estimation was used with 5000 samples (Hayes, 2018) to test the mediation effect, and a bias-corrected 95% CI was considered to determine the effects at the 2.5th and the 97.5th percentiles; when 0 was not included in the CI, the indirect effects were significant.

To assess the sample size for the SEM, including mediation effects via Sexual Esteem, a Monte Carlo simulation was conducted in Mplus. Assuming a medium-sized population effect (β = 0.30), 5000 replications were performed with a sample size of N = 400. The results indicated that the proportion of significant effects, including indirect effects (α = 0.05), achieved satisfactory power levels (i.e., >0.80). The results indicated that the empirical standard errors (S.E.) for the focal paths ranged from 0.047 to 0.052, with 95% confidence interval coverage close to the nominal value. Since we set a medium-sized effect (β = 0.30), a further check was provided for the smaller effect and the null effect. For the smaller effect, the observed standard error (approximately 0.05) indicates that the study was sufficiently powered to detect effects of this magnitude. For the null path, which was estimated near zero in the fitted model, the post hoc simulation yielded a proportion of significant replications close to 0.05, as expected under a true null effect. This pattern indicates that the model maintained appropriate Type I error rates and did not falsely identify spurious associations.

## 3. Results

### 3.1. Descriptive Results

Table 1 shows the reliability, mean scores, and *t*-tests for the scale scores. *t*-tests showed significant differences between men and women for only the BAS (t (460.81) = 6.10, *p* < 0.001; Cohen’s d = 0.56) and SEX-EST (t (470) = 3.33, *p* < 0.001; Cohen’s d = 0.31). Gender was recoded in a dummy variable (0 = Men; 1 = Women). Those participants who responded “other” were excluded from this new variable.

In both cases men showed higher scores (BAS = 3.66; sd = 0.75; SEX-EST = 3.47; sd = 0.93) compared to women (BAS = 3.21, sd = 0.86; SEX-EST = 3.19, sd = 0.92). Bivariate correlations are presented in Table 2.

Results revealed that SAQ-COM and SAQ-REF were positively correlated with BAS, SEX-EST and DFRS. BAS was positively correlated with SEX-EST and DFRS, and SEX-EST was also positively correlated with DFRS. Relationship duration was negatively correlated with SEX-EST, and the dummy variable men and women (M = 0) was negatively correlated with SEX-EST and DFRS. In line with original studies, Cronbach’s alpha confirmed good reliability for each scale, and CFA showed good fit criteria and a factorial structure. Model results for CFAs are presented in Supplementary Materials.

### 3.2. Hypothesis Testing

**Figure 1:**
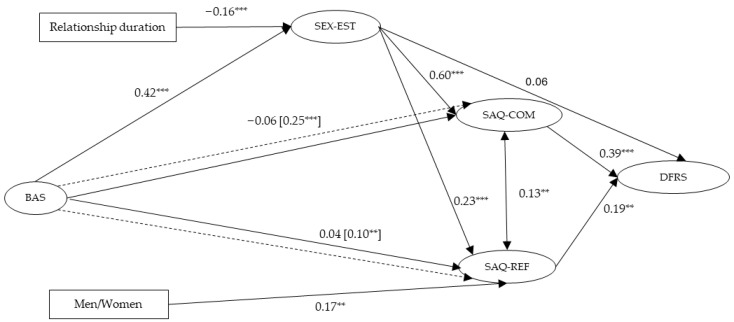
Standardized model results. Note. Indirect effects in dashed lines and standardized results in brackets. For control variables (Relationship duration and Men/Women), only significant associations were displayed for clarity. BAS: Body Appreciation scale; SEX-EST: Sexual esteem; SAQ-COM: the ability to initiate and communicate about sex; SAQ-REF: the ability to refuse unwanted sex; DFRS = Relationship satisfaction *** *p* < 0.001; ** *p* < 0.01.

The SEM was performed by entering BAS as the predictor for SEX-EST, SAQ-COM and SAQ-REF. SEX-EST was the mediator between BAS and SAQ-COM and SAQ-REF, while DFRS was the final outcome. Results are presented in Table 3 and Figure 1.

The model fit was good—χ^2^ (681) = 1095.81, *p* < 0.05; RMSEA = 0.036, 90% CI [0.032, 0.040]; CFI = 0.916; SRMR = 0.064—and results partially confirmed the hypotheses.

H1 was not confirmed, as the associations between BAS and SAQ-COM and BAS and SAQ-REF were not significant. H2 and H3 were confirmed because the positive association between BAS and SEX-EST was significant, and the mediating effect of SEX-EST was significant for both SAQ-COM and SAQ-REF. H4 was not confirmed because SEX-EST was not significantly associated with DFRS, while H5 was confirmed since SAQ-COM and SAQ-REF were both positively associated with DFRS.

Among the control variables, relationship duration was negatively associated only with SEX-EST, while Men/Women (M = 0) was positively associated only with SAQ-REF.

The model explained 34.0% of the variance for SAQ-COM, 9.6% for SAQ-REF, and 26.3% for DFRS.

To test H6, a structural invariance was performed to compare model results between women and men. To test structural invariance, all scales achieved measurement invariance. Specifically, full measurement invariance was reached for BAS, while partial measurement invariance was established for the other scales. SAQ reached partial invariance at the metric and scalar levels, SEX-EST at the scalar level, and DFRS at metric level.

All parameters released following the inspection of Modification Indices (MIs) were subsequently retained for the assessment of structural invariance. Results for the measurement invariance are presented in the Supplementary Materials.

Structural invariance between women and men was reached, confirming H6. In fact, no significant differences emerged in the relationships between variables, as reported in Table 4.

## 4. Discussion

The aim of the present study was to examine the factors associated with relationship satisfaction in a sample of individuals engaged in heterosexual romantic relationships. Specifically, we examined the role of body appreciation, sexual esteem, and sexual assertiveness. Although previous research has clarified the dyadic relationships between body appreciation, sexual esteem, and sexual assertiveness (Grower & Ward, 2018; Linardon et al., 2022; May & Johnston, 2022), to our knowledge, no studies have simultaneously examined the interrelationships between these three constructs and their combined role in romantic heterosexual relationship satisfaction. Here, we focused primarily on the role of sexual assertiveness, as previous studies have extensively documented its psychological and psychosocial benefits (Boket et al., 2016; Brassard et al., 2015; Loshek & Terrell, 2015; Moyano et al., 2021; Zhang et al., 2022), but its specific role in predicting relationship satisfaction in stable romantic relationships has not yet been fully explored.

### 4.1. Interpretation of Hypothesis Testing

The hypotheses based on the model that included the constructs under study were partially confirmed. Contrary to H1, body appreciation was not significantly associated with either dimension of sexual assertiveness (the ability to initiate and communicate about sex and the ability to refuse unwanted sex). Body appreciation, as a general acceptance of one’s body, is an important aspect in fostering sexual well-being (Linardon et al., 2022) but may not be sufficient to promote assertive sexual behaviors, which may require interpersonal competence, experience and a supportive relationship context. In other words, people may have a positive self-evaluation of their bodies but still lack the communication skills, confidence or emotional security to be assertive in sexual contexts.

In support of this explanation, H2 and H3 were confirmed. In fact, body appreciation was positively related to sexual esteem, which in turn mediated the relationship between body appreciation and both dimensions of sexual assertiveness. Thus, the ability to communicate sexual preferences and/or refuse unwanted sex may be related to body appreciation, but only when a general positive self-evaluation of sexual activity is included. These findings are consistent with those of previous studies (Grower & Ward, 2018; Linardon et al., 2022; May & Johnston, 2022), which found that positive body image contributes to greater self-confidence and self-perception in sexual contexts.

Regarding relationship satisfaction, H4 was not confirmed, as sexual esteem was not significantly associated with relationship satisfaction. It is possible that sexual esteem may influence individual experiences of sexuality to a greater extent than global evaluations of the relationship. Sexual esteem, as conceptualized, emphasizes a strictly individual evaluation of the self as a sexual being, with a strong emphasis on the individual perspective (Brassard et al., 2015). One possible explanation is that sexual esteem represents an intrapersonal self-evaluation, whereas sexual assertiveness reflects an interpersonal behavioral competence that is more proximal to relationship processes. Assertiveness may directly affect communication and boundary-setting within the couple, which are critical for relational dynamics and satisfaction (Bennett-Brown & Denes, 2023; Mallory, 2022). Relationship satisfaction may instead depend not solely on sexual activity, but rather on a multitude of factors, including relational dynamics, emotional regulation, and the duration of the relationship (Bühler et al., 2020; Rollero, 2022). In the present study, the relationship duration plays a role in sexual esteem solely. The findings suggest that prolonged relationships may appear to diminish an individual’s sexual self-esteem. In addition, the relevance of communication in a relationship may partially explain why H5 was confirmed, as both sexual assertiveness dimensions were positively associated with relationship satisfaction. This result is consistent with previous research showing that open sexual communication and the ability to set sexual boundaries contribute to greater intimacy and relationship satisfaction (Beaulieu et al., 2023; Gimmel et al., 2024).

Finally, an additional aim of this study was to confirm the extent to which this model can be similar between men and women. This question arises in particular with regard to sexual assertiveness, which is still primarily studied in women’s samples (Gil-Llario et al., 2022). The results (H6) did not show significant differences between men and women in the relationships between the variables. The mechanisms underlying the relationships between body appreciation, sexual esteem, sexual assertiveness and relationship satisfaction seem to be similar for men and women, even if some differences emerged in the ability to refuse unwanted sex, which seems more relevant for women than for men.

### 4.2. Limitations and Future Directions

Despite the contribution that the present study offers in understanding the factors that may promote relationship satisfaction, it is not exempt from limitations. First, the sample, as a convenience sample collected from a university context, is predominantly composed of Italian young adults and exclusively of heterosexual individuals in a stable relationship. This sample specificity limits the generalizability of the results. In fact, the findings derived from this preliminary study exploring the relationships among body appreciation, sexual assertiveness, and relationship satisfaction must be interpreted with caution. It is important to note that this study involves participants who are primarily young adults and whose relationships are still in their early stages, thus potentially exhibiting different relational dynamics compared to those observed in long-term adult relationships. Future research should include sexual and gender minorities to test the model’s generalizability across diverse populations. In addition, despite the work having implications for couples’ well-being, the present study could only investigate the perspectives of single individuals currently involved in a heterosexual relationship. Future studies should investigate the relationships tested in this study in dyads (i.e., couples) and not solely in single individuals. Moreover, this study relies on self-report measures, which may introduce biases such as social desirability or inaccurate recall. These factors could affect the validity of the findings, as participants may underreport or overreport certain behaviors or attitudes. Future research should consider incorporating multi-method approaches, such as partner reports or behavioral observations, to reduce these potential biases. Also, shorter versions of the scales may help reduce biases of response set, especially for SAQ (Nagy et al., 2025). Finally, another limitation was related to the cross-sectional design that precludes causal inferences. Longitudinal studies are needed to assess the temporal dynamics and potential causal pathways among the variables.

### 4.3. Practical Implications

The present study offers some insights into understanding what promotes relationship satisfaction. The findings highlight the importance of good sexual communication and positive body image, thereby providing a foundational framework for sexual affective education programs. The promotion of sexual assertiveness and body appreciation can facilitate the development of young people’s understanding of factors conducive to the quality of romantic relationships, as well as the prevention of gender stereotypes and rigid sexual scripts. Such educational programs may also be effective in preventing adverse dynamics such as coercion and emotional, sexual, and domestic violence.

## Figures and Tables

**Table 1:** Descriptive results of study variables and *t*-tests between men and women.

	M (SD)	M_men_ (DS)	M_women_ (DS)	Range	α	*t*-Test (df)	Cohen’s d
SAQ-COM	5.52 (1.16)	5.58 (1.01)	5.45 (1.29)	1–7	0.83	1.21 (443.28)	0.11
SAQ-REF	5.14 (1.37)	5.02 (1.37)	5.26 (1.37)	1–7	0.76	−1.90 (470)	0.17
BAS	3.43 (0.83)	3.66 (0.75)	3.21 (0.86)	1–5	0.93	6.10 *** (460.81)	0.56
SEX-EST	3.32 (0.94)	3.47 (0.93)	3.19 (0.92)	1–5	0.91	3.33 *** (470)	0.31
DFRS	3.11 (0.63)	3.14 (0.61)	3.08 (0.64)	0–4	0.89	1.04 (470)	0.09

Note. N_men_ = 237; N_women_ = 235. SAQ-COM: the ability to initiate and communicate about sex; SAQ-REF: the ability to refuse unwanted sex; BAS: Body Appreciation scale; SEX-EST: Sexual esteem; DFRS = Relationship satisfaction. *** *p* < 0.001.

**Table 2:** Bivariate correlations between scale scores.

	1	2	3	4	5	6	7
1. SAQ-COM	-						
2. SAQ-REF	0.26 ***	-					
3. BAS	0.15 ***	0.11 *	-				
4. SEX-EST	0.50 ***	0.22 ***	0.41 ***	-			
5. DFRS	0.42 ***	0.26 ***	0.17 ***	0.32 ***	-		
6. Relationship duration	−0.09	−0.08	0.003	−0.16 ***	−0.05	-	
7. Men/Women	−0.06	0.08	−0.27 ***	−0.15 ***	−0.05	-	-

Note. SAQ-COM: the ability to initiate and communicate about sex; SAQ-REF: the ability to refuse unwanted sex; BAS: Body Appreciation scale; SEX-EST: Sexual esteem; DFRS = Relationship satisfaction *** *p* < 0.001; * *p* < 0.05. Correlation coefficients involving the dichotomous Men/Women variable (0 = Men; 1 = Women) represent point-biserial correlations.

**Table 3:** Model results.

Paths	Β	*B*	(SE)	BC 95% CI
Direct effects				
BAS→SEX-EST	0.42 ***	0.47 ***	(0.05)	[0.37, 0.58]
BAS→SAQ-COM	−0.06	−0.08	(0.05)	[−0.19, 0.03]
BAS→SAQ-REF	0.04	0.04	(0.05)	[−0.06, 0.15]
SEX-EST→SAQ-COM	0.60 ***	0.65 ***	(0.06)	[0.52, 0.79]
SEX-EST→SAQ-REF	0.23 ***	0.21 ***	(0.05)	[0.12, 0.31]
SEX-EST→DFRS	0.06	0.06	(0.06)	[−0.06, 0.17]
SAQ-COM→DFRS	0.39 ***	0.36 ***	(0.06)	[0.26, 0.48]
SAQ-REF→DFRS	0.19 ***	0.20 ***	(0.05)	[0.10, 0.31]
Men/Women→SEX-EST	0.01	0.01	(0.08)	[−0.17, 0.19]
Relationship duration→SEX-EST	−0.16 ***	−0.004 ***	(0.001)	[−0.006, −0.003]
Men/Women→SAQ-COM	0.00	0.001	(0.08)	[−0.20, 0.20]
Relationship duration→SAQ-COM	−0.03	−0.001	(0.001)	[−0.003, 0.001]
Men/Women→SAQ-REF	0.17 ***	0.35 ***	(0.09)	[0.18, 0.54]
Relationship duration→SAQ-REF	−0.08	−0.002	(0.001)	[−0.003, 0.000]
Men/Women→DFRS	−0.05	−0.11	(0.08)	[−0.29, 0.07]
Relationship duration→DFRS	0.04	0.001	(0.001)	[−0.001, 0.003]
Indirect effects				
BAS→SEX-EST→SAQ-COM	0.25 ***	0.31 ***	(0.05)	[0.22, 0.40]
BAS→SEX-EST→SAQ-REF	0.10 **	0.10 **	(0.03)	[0.05, 0.15]

Note. BAS: Body Appreciation scale; SEX-EST: Sexual esteem; SAQ-COM: the ability to initiate and communicate about sex; SAQ-REF: the ability to refuse unwanted sex; DFRS = Relationship satisfaction. Β = Standardized results; *B* = Unstandardized results; BC 95% CI = Bias-Corrected 95% Confidence Interval. *** *p* < 0.001; ** *p* < 0.01.

**Table 4:** Model fit for structural invariance.

	χ^2^	df	RMSEA	CFI	SRMR	Δχ^2^ (df)	ΔRMSEA	ΔCFI	ΔSRMR
M1 (baseline)	1795.38 *	1421	0.033	0.907	0.072	-	-	-	-
M2 (structural)	1827.18 *	1438	0.033	0.906	0.078	29.58 * (17)	0.000	−0.001	0.006

Note. * *p* < 0.05.

## Data Availability

The raw data supporting the conclusions of this article will be made available by the authors upon request.

## Supplementary Materials

The following supporting information can be downloaded at https://www.mdpi.com/article/10.3390/bs15121730/s1, Table S1. Model fit for CFA. Table S2. Standardized loadings for CFAs. Table S3. Measurement invariance.
