# Supplementary material for: Satisfaction in Romantic Relationships: The Role of Body Appreciation, Sexual Esteem and Sexual Assertiveness
Source: Behav Sci (Basel). 2025 Dec 15;15(12):1730. doi: 10.3390/bs15121730 (PMC12730133; doi:10.3390/bs15121730)
Supplement: Supplementary file 1 [file behavsci-15-01730-s001.zip › behavsci-3939043-supplementary.pdf]

Table S1. Model fit for CFA

	$\chi 2$ (df)	RMSEA	CFI	SRMR
SAQ	226.99*** (62)[a]	0.075 (0.065,0.086)	0.900	0.071
BAS	141.41*** (35)	0.080 (0.67, 0.094)	0.958	0.037
SEX-EST	2.30 (3)[b]	0.000 (0.000, 0.070)	1.000	0.007
DFRS	90.37*** (27)	0.070 (0.055, 0.087)	0.952	0.039

Note. SAQ: Sexual assertiveness questionnaire; BAS: Body Appreciation scale; SEX-EST: Sexual esteem; DFRS = Relationship satisfaction ***p <.001. [a] = two residual covariances free; [b] = two residual covariances free

Table S2. Standardized loadings for CFAs

Sexual assertiveness questionnaire (SAQ)	SAQ-com	SAQ-ref
SAQ1. I feel uncomfortable telling my partner what feels good (R)	.53	
[Mi sento a disagio quando dico al mio partner cosa mi piace]		
SAQ2. I feel uncomfortable talking during sex (R)	.42	
[Parlare durante il sesso mi fa sentire a disagio]		
SAQ 3. I am open with my partner about my sexual needs	.71	
[Parlo apertamente con il mio partner dei miei bisogni sessuali]		
SAQ4. I let my partner know if I want to have sex	.80	
[Faccio sapere al mio partner se voglio fare sesso]		
SAQ5. I feel shy when it comes to sex (R)	.52	
[Quando si arriva a fare sesso, mi sento timido/a]		
SAQ6. I approach my partner for sex when I desire it	.73	
[Mi faccio avanti con il mio partner per fare sesso quando lo desidero]		
SAQ7. I begin sex with my partner if I want to	.49	
[Incomincio io a fare sesso con il mio partner se lo voglio]		
SAQ8. It is easy for me to discuss sex with my partner	.69	
[Per me è facile parlare di sesso con il mio partner]		
SAQ9. refuse to have sex if I don't want to		.43
[Mi rifiuto di fare sesso se non voglio]		
SAQ10. I find myself having sex when I do not really want it (R)		.60
[Mi sono ritrovato/a a fare sesso quando in realtà non lo volevo fare]		

SAQ11. I give in and kiss if my partner pressures me, even if I already said no (R)	.69
[Se il mio partner insiste, cedo e lo bacio, anche se gli/le avevo già detto di no]	
SAQ12. I have sex if my partner wants me to, even if I don't want to (R)	.93
[Faccio sesso con il mio partner se lui/lei vuole che lo faccia, anche se io non voglio]	
SAQ13. It is easy for me to say no if I don't want to have sex	.48
[Per me è facile dire di no se non voglio fare sesso]	
Residual covariances	
SAQ6 with SAQ7	.48
SAQ1 with SAQ2	.41
Correlations between factors	
SAQ-com with SAQ-ref	.23

Note. SAQ-com = the ability to initiate and communicate about sex; SAQ-ref = the ability to refuse unwanted sex. In brackets [] Italian translation. (R) reverse item. Residual covariances were relaxed after the inspection of the MIs.

Body appreciation scale (BAS)	
BAS1. I respect my body	.71
[Rispetto il mio corpo]	
BAS2. I feel good about my body	.88
[Mi sento bene con il mio corpo]	
BAS3. I feel that my body has at least some good qualities	.75
[Sento che il mio corpo ha qualche buona qualità]	
BAS4. I take a positive attitude toward my body	.87
[Ho un atteggiamento positivo verso il mio corpo]	
BAS5. I am attentive to my body's needs	.53
[Sono attento/a ai bisogni del mio corpo]	
BAS6. I feel love for my body	.85
[Provo amore per il mio corpo]	
BAS7. I appreciate the different and unique characteristics of my body	.76
[Apprezzo le caratteristiche diverse ed uniche del mio corpo]	
BAS8. My behavior reveals my positive attitude toward my body; for example, I walk holding my head high and smiling	.65

[Il mio comportamento rivela il mio atteggiamento positivo verso il mio corpo; per esempio, tengo la testa alta e sorrido]	
BAS9. I am comfortable in my body	.85
[Sono a mio agio nel mio corpo]	
BAS10. I feel like I am beautiful even if I am different from media images of attractive people (e.g., models, actresses/actors).	.77
[Mi sento di essere bello/a anche se sono diverso/a dalle immagini di persone attraenti proposte dai media (ad esempio modelli/e, attori/attrici)]	

Note. In brackets [] Italian translation from validation study (Casale et al., 2021).

The Sexual Esteem Subscale of the Multidimensional Sexuality Scale (SEX-EST)	
SEX-EST1. I am confident about myself as a sexual partner	.77
[Sono sicuro/a di me stesso come partner sessuale]	
SEX-EST2. I am a pretty good sexual partner	.82
[Sono un/una partner sessuale abbastanza buono/a]	
SEX-EST3. I am better at sex than most other people	.71
[Sono più bravo/a della maggior parte delle persone a fare sesso]	
SEX-EST4. I would rate myself pretty favorably as a sexual partner	.91
[Mi valuterei abbastanza bene come partner sessuale]	
SEX-EST5. would be very confident in a sexual encounter	.85
[Sarei molto sicuro/a di me in un rapporto sessuale]	
Residual covariances	
SEX-EST1 with SEX-EST2	.50
SEX-EST1 with SEX-EST5	.30

Note. In brackets [] Italian translation. Residual covariances were relaxed after the inspection of the MIs.

Dyadic-Familiar Relationship Satisfaction Scale (DFRS)	
DFRS1. Way in which my desires and needs are satisfied within my current relationship	.66
[Modo in cui i miei desideri e bisogni sono stati soddisfatti nell'attuale relazione]	
DFRS2. Stability of my couple relationship	.69

[Stabilità dell'attuale relazione di coppia]	
DFRS3. Way in which my partner and I make decisions concerning our couple life	.79
[Modalità con cui io e il/la mio/a partner prendiamo decisioni che riguardano la vita di coppia]	
DFRS4. Sharing of activities, rules, meanings, and values	.73
[Condivisione di attività, regole, significati, valori]	
DFRS5. Reciprocal comprehension	.81
[Comprensione reciproca]	
DFRS6. Reciprocal respect	.67
[Rispetto reciproco]	
DFRS7. Support I receive from my partner	.72
[Sostegno che ricevo dal/dalla partner]	
DFRS8. Quality of sexuality	.45
[Qualità della sessualità]	
DFRS9. Solution of our conflicts	.69
[Soluzione dei nostri conflitti]	

Note. In brackets [] Italian translation from original study (Raffagnino & Matera, 2015)

Table S3. Measurement invariance

Body Appreciation Scale (BAS) 93

	χ^2	df	RMSEA	CFI	SRMR	Δ χ^2	Δ df	ΔRMSEA	ΔCFI	ΔSRMR
Configural invariance	145.76*	68	0.070	0.967	0.035	-	-	-	-	-
Metric invariance	163.31*	77	0.069	0.963	0.059	19.48*	9	-0.001	-0.004	0.024
Scalar invariance	184.92*	86	0.070	0.958	0.063	25.65	9	0.001	-0.005	0.004

Sexual Assertiveness Questionnaire (SAQ)

	χ^2	df	RMSEA	CFI	SRMR	Δ χ^2	Δ df	ΔRMSEA	ΔCFI	ΔSRMR
Configural invariance	249.01*	124	0.065	0.917	0.075	-	-	-	-	-
Metric invariance	274.63*	135	0.066	0.907	0.086	31.08	11	0.001	-0.010	0.011
Metric invariance[a]	266.39*	134	0.065	0.912	0.084	20.10*	10	0.000	-0.005	0.009
Scalar invariance	347.98*	145	0.077	0.864	0.094	113.69*	11	0.012	-0.048	0.010
Scalar invariance[b]	278.58*	142	0.064	0.909	0.085	14.54	8	-0.001	-0.003	0.001

Sexual Esteem (SEX-EST)

	χ^2	df	RMSEA	CFI	SRMR	Δ χ^2	Δ df	ΔRMSEA	ΔCFI	ΔSRMR
Configural invariance	2.79	6	0.000	1.000	0.007	-	-	-	-	-
Metric invariance	9.81	12	0.000	1.000	0.042	7.32	6	0.000	0.000	0.035
Scalar invariance	26.02	16	0.052	0.992	0.053	20.26*	4	0.052	-0.008	0.011
Scalar invariance[c]	14.70	15	0.000	1.000	0.044	5.58	3	0.000	0.000	0.002

Relationship Satisfaction (DFRS)

Configural invariance	133.29*	54	0.079	0.936	0.044	-	-	-	-	-
Metric invariance	149.62*	62	0.077	0.930	0.065	18.21*	8	-0.002	-0.006	0.021
Metric invariance[d]	143.86*	61	0.076	0.934	0.058	11.12	7	-0.003	-0.002	0.014
Scalar invariance	154.20*	69	0.072	0.932	0.060	10.23	8	-0.004	-0.002	0.002

Note. *p<. 05. SAQ: a = Free loading on item SAQ10 after the inspection of MIs. b= free intercept on SAQ7, SAQ9, SAQ, 10 after the inspection of MIs ; SEX-EST c= free intercept on SEX-EST3 after the inspection of MIs; DFRS d= free loading on DFRS5 after the inspection of MIs.
BAS: Body Appreciation scale; SEX-EST: Sexual esteem; SAQ-com: the ability to initiate and communicate about sex; SAQ-ref: the ability to refuse unwanted sex; DFRS = Relationship satisfaction
